# Association between Dietary Habits and Sarcopenia in Patients with Liver Cirrhosis

**DOI:** 10.3390/jcm12144693

**Published:** 2023-07-14

**Authors:** Mirabela-Madalina Topan, Ioan Sporea, Mirela Dănilă, Alina Popescu, Ana-Maria Ghiuchici, Raluca Lupușoru, Roxana Șirli

**Affiliations:** 1 Department of Gastroenterology and Hepatology, “Victor Babeș” University of Medicine and Pharmacy, 300041 Timisoara, Romania; mirabelamadalina1990@gmail.com (M.-M.T.); isporea@umft.ro (I.S.); alinamircea.popescu@gmail.ro (A.P.); anamaria.ghiuchici@gmail.com (A.-M.G.); raluca_lupusoru@yahoo.ro (R.L.); roxanasirli@gmail.com (R.Ș.); 2 Advanced Regional Research Center in Gastroenterology and Hepatology, “Victor Babeș” University of Medicine and Pharmacy, 300041 Timișoara, Romania; 3 Center for Modeling Biological Systems and Data Analysis, Department of Functional Science, “Victor Babeș” University of Medicine and Pharmacy, 300041 Timișoara, Romania

**Keywords:** sarcopenia, liver cirrhosis, dietary habits, sarcopenic obesity

## Abstract

Sarcopenia and sarcopenic obesity are frequent complications of cirrhosis, and the dietary patterns of patients with these diseases significantly impact the development of both conditions. This study aims to evaluate the adequacy of the dietary intake of patients with liver cirrhosis. A total of 201 patients with liver cirrhosis were included in this analysis. We evaluated the nutritional status of the patients as stated by EWGSOP2 criteria. Subjects were divided into three groups: non-sarcopenic, sarcopenic, and with sarcopenic obesity. We conducted a dietary assessment three times over nonconsecutive 24 h periods within a month. According to EWGSOP2 criteria, combining low handgrip strength with low skeletal muscle index, the prevalence of sarcopenia was 57.2%. Sarcopenic obesity was found in 25.3% of patients. In the sarcopenic group of patients, the energy intake was lower than the current recommendations. Regarding protein intake, the consumption was relatively low in both sarcopenia and sarcopenic obesity samples of patients (0.85 g/kg body weight and 0.77 g/kg BW, *p* < 0.0001). Patients had a median of 2–3 eating episodes daily, and they often missed late-evening snacks. In conclusion, diet quality in cirrhotic patients was relatively poor, and energy and protein intakes were lower than suggested.

## 1. Introduction

*Liver cirrhosis* is an end-stage liver disease that has a variety of complications. Sarcopenia and sarcopenic obesity are frequent but often neglected complications in these patients. Sarcopenia affects 30–70% of them [1] and is related to an increase in morbidity and mortality.

In recent years, with the increasing number of obese cirrhotic patients, the prevalence of sarcopenic obesity has risen; it ranges from 20% to 35% and is also associated with increased mortality [2].

The European Working Group on Sarcopenia in Older People (EWGSOP2) Guideline on sarcopenia 2019 [3] concentrates on low muscle strength as a main characteristic of sarcopenia. It identifies low muscle quantity and quality to confirm sarcopenia diagnosis. Sarcopenic obesity means decreased muscle mass and function associated with increased fat mass. 

The limited knowledge of cirrhotic patients about the importance of adequate nutrition can influence food consumption through either reduced or excess intake, leading to undernutrition or obesity.

Insufficient dietary intake is an essential cause of sarcopenia in cirrhotic patients. In a study conducted by Campillo et al., inadequate dietary intake was a self-dependent predictor of in-hospital mortality, and a decrease in daily caloric intake was associated with worsening progressive liver failure [4]. Several international organizations have recently published recommendations concerning the quantity, variety, and distribution of macro- and micro-nutrients in patients with cirrhosis [5,6]. A brief synthesis of these recommendations is shown in Table 1.

Obese cirrhotic patients often have a deficiency in specific aspects of nutritional status. For example, they have poor protein intake and micronutrient deficits but excess daily caloric intake.

An investigation of dietary habits would be beneficial to unveil the differences with conventional nutritional recommendations and effectively enhance patient compliance with them. Limited studies have assessed total calorie intake and the components of total calories regarding protein, carbohydrates, and fat in patients with cirrhosis, so the present study aimed to estimate the effects of an improper oral diet on the nutritional status of these patients.

## 2. Materials and Methods

### 2.1. Population Selection

This prospective study was performed in a tertiary Department of Gastroenterology and Hepatology on 201 liver cirrhotic patients from January 2019 to December 2020.

Liver cirrhosis was diagnosed with a mix of laboratory tests, abdominal ultrasounds, ultrasound-based elastographies, upper endoscopies, and radiological evidence. We assessed the severity of cirrhosis using Child–Pugh’s score, the Model for End-Stage Liver Disease (MELD) score, and the albumin–bilirubin score (ALBI).

**Figure 1:**
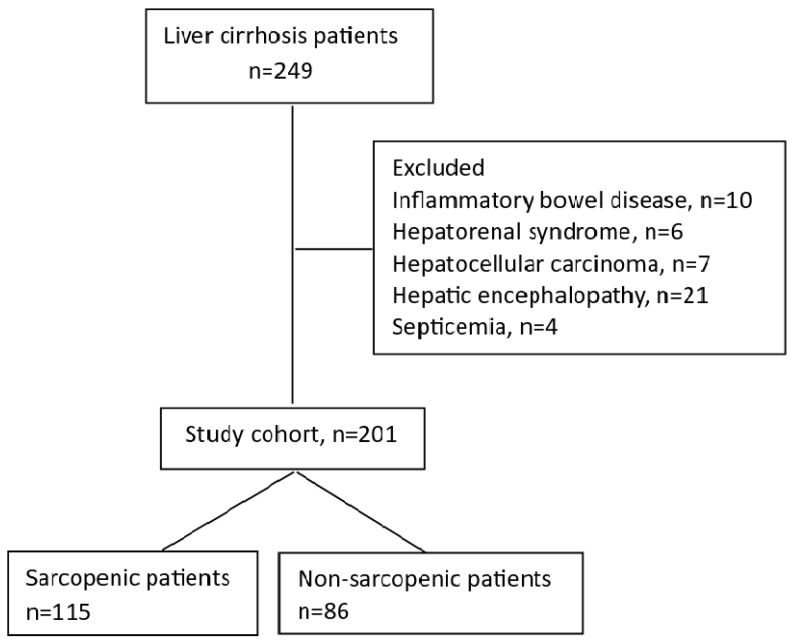
Study flowchart.

A total of 249 liver cirrhosis patients were enrolled in the study, but only 201 fulfilled the inclusion criteria and were included in the final analysis. Inclusion criteria were age greater than 18 years, liver cirrhosis, availability of a standard diagnostic method (contrast-enhanced CT and dynamometry), and dietary assessment. Exclusion criteria included patients with hepatic encephalopathy, pancreatic insufficiency, hepatorenal syndrome, coexisting human immunodeficiency virus, septicemia, tuberculosis, chronic renal failure, inflammatory bowel disease, enteral tube feeding, hepatocellular carcinoma, or other malignancies (Figure 1).

We designed the research protocol following the Helsinki Declaration of 1975. Every patient provided informed approval to participate in the study. The study was approved by the local Ethical Committee.

We assessed a dietary intake interview over three nonconsecutive 24 h periods for a month. We received guidance from a registered dietician on maintaining a detailed food record.

For all statistical analyses that were adjusted for the survey, we calculated the average sample weights for nutrient and dietary data for each day of the dietary interview.

### 2.2. Nutritional Assessment Tools

The body mass index (BMI) of all patients was determined by the equation weight/height2. Due to the prevalence of ascites and/or edema in patients with cirrhosis, we determined their dry weight (dry BMI) by subtracting a percentage of their actual weight based on the severity of their ascites. This percentage is 5% for mild cases, 10% for moderate cases, and 15% for severe cases, with an additional 5% for patients exhibiting bilateral pedal edema. We then categorized patients based on their BMI: those with a BMI < 18.5 kg/$m^{2}$ were considered undernourished, those with a BMI between 18.5–24.9 kg/$m^{2}$ were considered to have a normal weight, and those with a BMI > 25 kg/$m^{2}$ were considered overweight or obese. [5].

Handgrip strength (HGS): A Jamar dynamometer was used to measure dominant handgrip strength. The patient was seated with their arm resting alongside their body and the elbow bent at a 90-degree angle. Each patient completed the test three times using their dominant hand, with a 10–30 s break between the trials. All measurements were registered in kilograms. We used the following cut-off values: for men, an HGS < 27 kg was considered below average, while for women, an HGS < 16 kg was considered below average [3].

Skeletal muscle index (SMI): Muscle mass assessments were examined using cross-sectional skeletal CT images at the level of the lumbar three vertebrae (L3) by an expert radiologist using National Institutes of Health ImageJ (NIH ImageJ, V 1.8.0) software. The standard attenuation values for muscle tissue ranged from 29 to 150 Hounsfield units (HU). We normalized the cross-sectional areas based on each patient’s height. We also calculated the skeletal muscle index, defined as cross-sectional muscle area/${\mathrm{height}}^{2}$. The presence of low muscle mass was defined using the following cut-off values: SMI < 50 ${\mathrm{cm}}^{2}/m^{2}$ for men and 39 ${\mathrm{cm}}^{2}/m^{2}$ for women [5].

We used the EWGSOP2 criteria [3] to identify sarcopenia, which we defined as having low muscle strength based on handgrip strength and a low skeletal muscle index determined by contrast-enhanced CT. Sarcopenic obesity was defined as having both low sex-adjusted SMI and low HGS, as well as a BMI ≥ 25 kg/m^2^.

### 2.3. Assessment of Dietary Intake

Each patient had a nutrition assessment conducted by a registered dietitian. Dietary intake was determined by 24 h diet recall within a month interval, recognized by EASL as an optimal method to assess dietary in patients with cirrhosis [5]. This technique, proposed by the US Department of Agriculture, provides a quantitative and subjective analysis of food consumption during three nonconsecutive 24 h recalls (2 weekdays and 1 weekend day) [7]. Data were analyzed for energy and macronutrient intake using nutrition management software (Eat & Track V 1.8.1). Total energy (kcal), protein (g) intake, and percentage of carbohydrate and lipid intake were calculated accordingly. Patients were also asked about their vegetarian (fruits, vegetables) and non-vegetarian diets (fish, meat), the number of meals and snacks consumed in a day, and late evening snacks, sweets, dietary products, and alcohol consumption. The participants were asked to indicate how often they consumed each food item over the past year regarding the number of specified meal proportions consumed per day/weekly/occasionally/rarely/never.

To assess adherence, we compared the average daily intake of each patient to the latest nutritional guidelines recommendations.

### 2.4. Statistical Analysis

We used the MedCalc software for Windows (MedCalc Software, version 19.3.1, Ostend, Belgium) to make the statistical analysis. For testing the distribution of numerical variables, the Kolmogorov–Smirnov test was used. Qualitative variables were presented as numbers and percentages. To evaluate differences between numerical variables with normal distribution, parametric tests such as *t*-test and ANOVA were utilized. Nonparametric tests like Mann–Whitney or Kruskal–Wallis tests were used for variables with non-normal distribution. To determine the statistical significance of differences between proportions, chi-square test with Yates’ correction for continuity was utilized. Univariate and multivariate analysis was also conducted to observe factors associated with sarcopenia. We utilized a confidence level of 95% along with a significance level of 5%. It is important to note that all *p*-values were two-tailed.

## 3. Results

### 3.1. Patients’ Characteristics

The analysis involved 201 patients, whose mean age was 61.6 ± 9.4 years. Based on the Child–Pugh Classification, 20.4% (41/201) were A-class, 40.7% (82/201) were B, and 38.9% (78/201) were C. Regarding etiology, 55.2% (111/201) had alcoholic cirrhosis, 27.3% (55/201) had hepatitis C virus (HCV) cirrhosis, 12.9% (26/201) had hepatitis B virus (HBV) cirrhosis, and 4.6% (9/201) had other etiologies. Regarding the ALBI score, 57.2% (115/201) had a grade 3 score. The characteristics of the study population are presented in Table 2.

### 3.2. Prevalence of Sarcopenia and Sarcopenic Obesity

Based on the EWGSOP2 criteria, the prevalence of sarcopenia in our entire cohort was 57.2% (115/201) when low handgrip strength was combined with a low skeletal muscle mass index. Among the decompensated group, 97% (109/201) had sarcopenia, while only 3% (6/201) were sarcopenic in the compensated group.

Regarding sarcopenic obesity, 47.8% (96/201) patients had a BMI greater than 25, but only 25.4% (51/201) had sarcopenic obesity.

### 3.3. Nutrient Intake

We divided the population in our cohort into three groups: non-sarcopenic, sarcopenic, and sarcopenic obesity. A comparison of nutrient intakes between the three groups after being adjusted for age, sex, and weight is shown in Table 3.

Table 4 shows the prevalence of consumption frequency of food intake markers in individuals with cirrhosis divided into three groups.

In all three groups, the consumption of fruits was, on average, two or three portions per day. Regarding vegetables, in the sarcopenic obesity group, the consumption was once or twice a day, compared to the other two groups, where the consumption was higher: twice or three times a day. Sweets consumption was very high in the obese sarcopenic group (64.70% daily consumption) compared to the non-sarcopenic and sarcopenic groups (44.2% daily and 31.3% daily), with a significant difference (*p* < 0.001).

Compared with the sarcopenic groups, the average consumption of dairy products was high in the non-sarcopenic group (63.95% daily consumption compared to 7.8% daily in patients with sarcopenia). The difference was significant (*p* < 0.0001).

The weekly consumption of meat was relatively low in sarcopenic groups (approximately 50% only three times/week) compared with the non-sarcopenic group (more than 30% 6–7 times/week), with a significant difference (*p* < 0.0001). The same patterns could be seen regarding fish consumption.

Patients’ reported eating episodes, physical activity, and alcohol consumption are shown in Table 5.

The median of the reported eating episodes in our cohort was 2–3 meals/day. Regarding late-night snacks, 70% of the patients in the non-sarcopenic group had one late-night snack, as compared to the sarcopenic and sarcopenic obesity groups, in which 6.96% and 11.8% of patients, respectively, had one late-night meal.

The rate of sedentary behavior was high in our groups, with only 30.4% of the non-sarcopenic group exercising regularly vs. 2–6% in the sarcopenic groups.

We found a high rate of alcohol consumption in our sample of patients, with 60–70% of patients in the sarcopenic groups admitting to consumption of alcohol.

### 3.4. Dietary Patterns and Sarcopenia: Multivariate Analysis

Using multiple logistic regression analyses, we found risk factors associated with dietary habits for developing sarcopenia (Table 6).

Patients with low dairy consumption have a 20 times higher chance of developing sarcopenia; those who consume alcohol, 10 times higher; and those with low consumption of vegetables, 3 times higher. In contrast, high consumption of meat has a protective role, OR = 0.22.

### 3.5. Dietary Patterns and Sarcopenic Obesity: Multivariate Analysis

We conducted the same analysis as above for patients with sarcopenic obesity (Table 7), and we found out that obese patients with low dairy consumption have a 7.8 times higher chance of developing sarcopenia; those who consume alcohol, a 3.8 times higher chance; those with low consumption of vegetables, a 5.2 times higher chance; and those with high consumption of sweets have a 7.5 times higher chance, while high consumption of meat has a protective role, like in the sarcopenic group (OR = 0.10).

### 3.6. Mortality Associated with Sarcopenia and Sarcopenic Obesity in Cirrhotic Patients

We analyzed the mortality rates at 6 and 12 months with regard to nutritional status. The six-month mortality rate was significantly higher in sarcopenia vs. sarcopenic obesity: 26.31% (53/201) vs. 7.46% (15/201) (*p* < 0.0001). Furthermore, the twelve-month mortality rate was also significantly higher in sarcopenia vs. sarcopenic obesity: 46.26% (93/201) vs. 19.9% (40/201) (*p* < 0.0001). A patient with cirrhosis and sarcopenia is 11.5 times more likely to die at six months and 9.8 times more likely to die at one year than a non-sarcopenic cirrhotic patient. The mortality rate for non-sarcopenic patients was 6.9% at 6 months and 17.5% at 1 year.

## 4. Discussion

This study aimed to evaluate the dietary patterns of a selection of patients with cirrhosis of various etiologies and disease stages regarding quality and quantity and to assess their adequacy compared with the latest guidelines. Furthermore, energy and protein intake associations with anthropometric parameters and survival status were explored.

EASL [5] and ESPEN [6] recommend an energy intake of at least 35 kcal/kg BW. The average energy intake in our sarcopenic group was lower than the suggestions (32.52 ± 7.56 kcal/kg BW), but in the group of non-sarcopenic and sarcopenic obesity, the intake was in accordance with the recommendations. Regarding macronutrient intake, we found that protein intake was relatively low compared to the guidelines in the sarcopenic and sarcopenic obesity samples of patients (0.85 g/kg BW and 0.77 g/kg BW, *p* < 0.0001). The Kirrhos study [8] found that patients with cirrhosis did not meet the recommended energy and protein intake levels. These results are similar to our findings.

Concerning carbohydrates and simple carbohydrates intake, we discovered that the three groups had average carbohydrate consumption (corresponding to 48–57% of total energy intake) and high sugar intake (> 22% of total energy intake), especially in the sarcopenic obesity group. Our findings are similar to a study of Buscail et al., in which the average carbohydrate consumption was 44–47% of the total energy intake and the sugar intake was more than 20% of the total energy intake [9].

The proportion of lipid intake was higher than the recommendations in all three groups, but in the sarcopenic obesity group, the average lipid consumption was the highest (43.43 ± 8.03%). This, along with the high consumption of simple carbohydrates, can explain the fat mass covering the low muscle mass in these patients [10].

On a food group level, we found that in our sample of patients, the three groups had different dietary habits, which were linked to their anthropometric parameters. Sarcopenic patients registered low consumption of essential food groups, such as vegetables, dairy, meat, and fish. In contrast, high consumption of fats and sugars was recorded, especially in the obese sarcopenic group.

Our findings are similar to the Kirrhos study [8], which reported low consumption of non-refined cereals, legumes, fruits and vegetables, poultry, and fish but high consumption of red meat and sugars among patients with cirrhosis.

The three study groups had a relatively similar distribution of dietary intake during meals and snacks. However, the median of the reported eating episodes did not fulfill the recommended range of 4–6 meals/snacks per day, as recommended by ESPEN [6].

Concerning late-night snacks, only 6.96% and 11.8% of the patients in the sarcopenic and sarcopenic obesity groups, respectively, had one. A similar result was found in a study on patients with cirrhosis on an awaiting liver transplant list [11].

A meta-analysis conducted by C-J Chen in 2019 [12] showed that having a late-night snack can enhance liver function reserve for patients with liver cirrhosis. 

As for the environmental aspects, the rate of sedentary behavior was high in our groups, with only 2–6% of the sarcopenic group exercising regularly. It has been shown that low physical activity has been correlated with worsened clinical course of cirrhosis patients, leading to sarcopenia. [13]

We found a high rate of alcohol consumption in our sample of patients. A total of 60–70% of patients in the sarcopenic groups admitted to regular consumption of alcohol. This high proportion of alcohol consumption in our group can be explained by the fact that the main etiology of cirrhosis in our group is alcohol abuse. Furthermore, it is well known that ethanol reduces muscle protein synthesis and accelerates proteolysis, leading to sarcopenia.

Using multiple logistic regression analyses in our sample of sarcopenic patients, we found out that patients with low dairy product consumption have twenty times more risk of developing sarcopenia, those who consume alcohol have ten times more risk, and those with low consumption of vegetables have three times more risk. In contrast, high meat consumption has a protective role (OR = 0.22).

We conducted the same analysis as above for patients with sarcopenic obesity. We discovered that patients with low consumption of dairy products have 7.8 times more risk of developing sarcopenia, those who consume alcohol have 3.8 times more risk, those with low consumption of vegetables have 5.2 times more risk, and those with high consumption of sweets have 7.5 times more risk. In contrast, high meat consumption has a protective role, like in the sarcopenic group (OR = 0.10).

In a study recently made on a Korean population [14], it was found that the consumption of meat/fish/egg/vegetable food groups and total food intake were inversely associated with the prevalence of sarcopenia.

Regarding six-month and one-year mortality in our cohort, we found that cirrhotic patients with sarcopenia have a worse prognosis than non-sarcopenic patients, regardless of overall body weight or BMI. These findings are similar to those from a study published by A J. Montano-Loza on sarcopenic obesity [10], where the mortality rates in patients with sarcopenia or sarcopenic obesity were 1.5 to 2 times higher compared with non-sarcopenic patients.

This study has some limitations. It is important to note that the 24 h recall method may have limitations in accurately recalling and retrieving details about the food consumed the previous day. We tried to minimize this limitation by excluding patients with hepatic encephalopathy and by having a professional dietician who conducted the interviews. Another limitation of the study is the absence of measuring resting energy expenditure, which hinders an accurate evaluation of the patients’ daily energy requirements.

Despite these limitations, our study provides useful information regarding dietary habits among patients with cirrhosis regarding quality and quantity, which are currently lacking, especially in our country.

## 5. Conclusions

Correct eating habits are essential for the overall well-being of an individual. Nutrition is a major factor contributing to the course of sarcopenia setting in patients. In our group, the dietary behavior of patients with cirrhosis and sarcopenia significantly differed from non-sarcopenic subjects. The dietary standards fell short, and the energy and protein intake was below the suggested levels. Due to the increased risk of mortality associated with inadequate nutrition, patients diagnosed with cirrhosis should be treated in an interdisciplinary way in cooperation with a doctor and a dietician.

## Figures and Tables

**Table 1:** General nutritional recommendations for cirrhotic patients.

Nutritional Recommendations	
Total energy intake	35 kcal/kg of body weight
Protein	1.2–1.5 g/kg of body weight
Total carbohydrates	45–75% of caloric intake
Simple carbohydrates	10–15% of caloric intake
Lipids	20–30% of caloric intake

**Table 2:** Baseline characteristics of the 201 patients studied.

Parameter	Values
Age (years) (mean ± SD) • <40 years • 40–60 years • >60 years	61.6 ± 9.4 1 (0.5%) 81 (40.3%) 119 (59.2%)
Gender • Men (%) • Women (%)	127 (63.2%) 74 (36.8%)
Child–Pugh classification • A • B • C	41 (20.4%) 82 (40.7%) 78 (38.9%)
Mean Child–Pugh score (points)	8.7 ± 2.2
Mean MELD score (points)	16.5 ± 7.5
Ascites n (%) • Absent • Present	157 (78.10%) 44 (21.90%)
Etiology of cirrhosis n (%) • Hepatitis B • Hepatitis C • Alcohol abuse • Other	26 (12.93%) 55 (27.36%) 111 (55.22%) 9 (4.49%)
Mean BMI (kg/m^2^) • Underweight—n (%) • Normal weight—n (%) • Overweight—n (%)	24.5 ± 4.4 18 (8.95%) 87 (43.28%) 96 (47.77%)
Comorbidities	
Hypertension	125 (69.65%)
Diabetes mellitus	89 (44.2%)
Metabolic sindrome	35 (17.41%)
ALBI score	
ALBI grade 1	7 (3.50%)
ALBI grade 2	79 (39.30%)
ALBI grade 3	115 (57.20%)
Laboratory findings	
Albumin (mg/dL)	2.73 ± 0.84
Trombocites (mm^3^)	124,697 ± 69.80
Total bilirubin (mg/dL)	3.07 ± 1.84
INR	1.60 ± 0.46
CRP (mg/dL)	47.37 ± 6.08

Abbreviations: SD—standard deviation; MELD—model for end-stage liver disease; BMI—body mass index; CRP—C-reactive protein; INR—international normalized ratio.

**Table 3:** Comparison between the three groups regarding nutrient intake.

Parameter	Non-Sarcopenic	Sarcopenic	Sarcopenic Obesity	*p*-Value
Total energy intake (kcal/kg BW)	38.72 ± 9.14	32.52 ± 7.56	37.74 ± 7.16	<0.0001
Protein (g/kg BW)	1.63 ± 0.31	0.85 ± 0.21	0.77 ± 0.20	<0.0001
Carbohydrates (%)	52.38 ± 9.93	57.00 ± 10.79	48.03 ± 8.72	<0.0001
Simple carbohydrates (%)	22.03 ± 9.37	21.95 ± 9.56	27.64 ± 10.40	0.003
Lipids (%)	30.87 ± 9.67	34.39 ± 0.98	43.43 ± 8.03	<0.0001

**Table 4:** Comparison of food group intakes.

Food Group	Non Sarcopenic	Sarcopenic	Sarcopenic Obesity	*p*-Value
Fruits (portions/day)				
0	1 (1.18%)	0	0	0.25
1	11 (12.79%)	19 (16.52%)	4 (7.84%)	0.16
2	35 (40.69%)	54 (46.95%)	17 (33.34%)	0.10
3	33 (38.37%)	38 (33.04%)	26 (50.98%)	0.45
4	6 (6.97%)	4 (3.49%)	4 (7.84%)	0.22
Vegetables (portions/day)				
1	14 (16.27%)	31 (26.95%)	19 (37.25%)	0.004
2	27 (31.39%)	49 (42.60%)	28 (54.90%)	0.15
3	43 (50.00%)	34 (29.56%)	4 (7.85%)	0.01
4	2 (2.34%)	1 (0.89%)	0	<0.0001
Sweets				
No	6 (6.97%)	5 (4.36%)	1 (1.96%)	0.20
Occasionally	27 (31.39%)	44 (38.26%)	16 (31.38%)	0.39
Rarely	15 (17.44)	30 (26.08%)	1 (1.96%)	0.002
Daily	38 (44.20%)	36 (31.30%)	33 (64.70%)	0.0001
Dairy				
No	2 (2.34%)	21 (18.26%)	13 (25.49%)	<0.0001
Occasionally	24 (27.90%)	43 (37.39%)	19 (37.25%)	0.25
Rarely	5 (5.81%)	42 (36.52%)	15 (29.41%)	0.0002
Daily	55 (63.95%)	9 (7.83%)	4 (7.85%)	<0.0001
Meat (portions/week)				
1	0	2 (1.73%)	2 (3.93%)	0.08
2	1 (1.19%)	18 (15.65%)	9 (17.64%)	0.0004
3	3 (3.48%)	48 (41.73%)	27 (52.94%)	<0.0001
4	6 (6.97%)	38 (33.04%)	11 (21.56%)	0.01
5	14 (16.27%)	8 (6.95%)	2 (3.93%)	0.02
6	33 (38.37%)	1 (0.90%)	0	<0.0001
7	29 (33.72%)	0	0	<0.0001
Fish (portions/week)				
0	4 (4.65%)	34 (29.56%)	17 (33.33%)	<0.0001
1	14 (16.27%)	58 (50.43%)	30 (58.82%)	<0.0001
2	29 (33.72%)	19 (16.52%)	3 (5.88%)	<0.0001
3	29 (33.72%)	3 (3.49%)	1 (1.97%)	<0.0001
4	10 (11.64%)	0	0	<0.0001

**Table 5:** Patients’ reported eating episodes, physical activity, and alcohol consumption.

	Non Sarcopenic	Sarcopenic	Sarcopenic Obesity	*p*-Value
Number of meals/day				
1	0	3 (2.61%)	0	0.15
2	12 (13.95%)	51 (44.34%)	11 (21.56%)	<0.0001
3	61 (70.93%)	58 (50.44%)	37 (72.54%)	0.002
4	13 (15.12%)	3 (2.61%)	3 (5.90%)	0.001
Snacks/day				
0	1 (1.17%)	33 (28.69%)	5 (9.80%)	<0.0001
1	33 (38.37%)	63 (54.78%)	30 (58.82%)	0.02
2	47 (54.65%)	17 (14.78%)	14 (27.45%)	0.03
3	5 (5.81%)	2 (1.75%)	2 (4.23%)	0.15
Late-night snacks				
0	16 (18.61%)	107 (93.04%)	45 (88.20%)	<0.0001
1	70 (81.39%)	8 (6.96%)	6 (11.80%)	<0.0001
Physical activity				
0	60 (69.76%)	108 (93.91%)	50 (98.00%)	<0.0001
1	26 (30.24%)	7 (6.09%)	1 (2.00%)	<0.0001
Alcohol consumption				
No	64 (74.41%)	43 (37.39%)	15 (29.41%)	<0.0001
Yes	22 (25.59%)	72 (62.61%)	36 (70.59%)	<0.0001

**Table 6:** Multiple logistic regression analyses examining the association between dietary patterns and sarcopenia in cirrhotic patients.

Parameter	Univariate Analysis		Multivariate Analysis	
	OR 95% CI	*p*-Value	OR 95% CI	*p*-Value
Low consumption of dairy products	20.89 (9.29–46.99)	<0.0001	14.39 (3.38–61.14)	0.0003
Alcohol consumption	9.87 (4.80–18.20)	<0.0001	4.64 (1.28–16.80)	0.01
High consumption of meat	0.22 (0.10–0.56)	<0.0001	0.24 (0.22–0.65)	0.005
High consumption of sweets	0.57 (0.32–1.02)	0.06	NA	NA
Low physical activity	1.80 (1.23–5.26)	<0.0001	1.25 (0.89–3.25)	0.01
Fewer meals per day	1.56 (1.00- 2.54)	<0.0001	1.25	0.001
Low consumption of vegetables	3.04 (1.05–5.20)	0.003	2.20 (1.00–4.56)	0.01
High rate for in-hospital stay	1.42 (1.29–1.57)	<0.0001	1.38 (1.19–1.60)	<0.0001
MELD score	1.56 (1.00–1.98)	<0.0001	0.99 (0.78–1.10)	0.04
Child–Pugh score	1.01 (0.98–1.05)	0.03	1.02 (1.00–1.40)	0.002
ALBI score	1.25 (1.10–2.10)	<0.0001	1.00 (0.65–2.15)	0.01
CRP	1.02 (1.00–1.62)	<0.001	1.00 (0.98–1.78)	0.004

**Table 7:** Multiple logistic regression analyses examining the association between dietary patterns and sarcopenic obesity in cirrhotic patients.

Parameter	Univariate Analysis		Multivariate Analysis	
	OR 95% CI	*p*-Value	OR 95% CI	*p*-Value
Low consumption of dairy products	7.83 (2.68–22.87)	<0.0001	3.96 (1.04–15.09)	0.04
Alcohol consumption	3.80 (1.91–7.56)	<0.0001	1.56 (0.58–4.14)	0.36
High consumption of meat	0.10 (0.04–0.20)	<0.0001	0.06 (0.01–0.22)	<0.0001
High consumption of sweets	7.52 (2.51–11.54)	<0.0001	4.66 (1.61–14.11)	0.004
Low physical activity	4.25 (2.45–9.45)	<0.0001	3.05 (0.89–4.15)	0.15
Fewer meals per day	1.46 (0.86–2.47)	0.15	NA	
Low consumption of vegetables	5.21 (1.01–7.20)	0.02	3.41 (1.05–10.20)	0.001
High rate for in-hospital stay	1.10 (1.04–1.16)	<0.0001	1.05 (0.97–1.14)	<0.0001
MELD score	0.89 (0.52–1.20)	0.07	NA	
Child–Pugh score	1.02 (0.99–1.10)	0.10	NA	
ALBI score	1.01 (1.00–1.75)	0.24	NA	
CRP	1.35 (0.99–3.40)	<0.0001	1.40 (1.10–1.78)	0.003

## Data Availability

Not applicable.
